# A dimeric fluorescent protein yields a bright, red-shifted GEVI capable of population signals in brain slice

**DOI:** 10.1038/s41598-018-33297-y

**Published:** 2018-10-12

**Authors:** Bumjun Yi, Bok Eum Kang, Sungmoo Lee, Sophie Braubach, Bradley J. Baker

**Affiliations:** 1 The Center for Functional Connectomics, Korea Institute of Science and Technology, Seoul, Republic of Korea; 2 Division of Bio-Medical Science and Technology, KIST School, Korea University of Science and Technology (UST), Seoul, Republic of Korea; 3 Department of Transdisciplinary Studies, Graduate school of Convergence Science and Technology, Seoul National University, Suwon, Republic of Korea

## Abstract

A bright, red-shifted Genetically Encoded Voltage Indicator (GEVI) was developed using a modified version of the fluorescent protein, tdTomato. Dimerization of the fluorescent domain for ArcLight-type GEVIs has been shown to affect the signal size of the voltage-dependent optical signal. For red-shifted GEVI development, tdTomato was split fusing a single dTomato chromophore to the voltage sensing domain. Optimization of the amino acid length and charge composition of the linker region between the voltage sensing domain and the fluorescent protein resulted in a probe that is an order of magnitude brighter than FlicR1 at a resting potential of −70 mV and exhibits a ten-fold larger change in fluorescence (ΔF) upon 100 mV depolarization of the plasma membrane in HEK 293 cells. Unlike ArcLight, the introduction of charged residues to the exterior of dTomato did not substantially improve the dynamic range of the optical signal. As a result, this new GEVI, Ilmol, yields a 3-fold improvement in the signal-to-noise ratio compared to FlicR1 despite a smaller fractional change in fluorescence of 4% per 100 mV depolarization of the plasma membrane. Ilmol expresses well in neurons resolving action potentials in neuronal cultures and reporting population signals in mouse hippocampal acute brain slice recordings. Ilmol is the brightest red-shifted GEVI to date enabling imaging with 160-fold less light than Archon1 for primary neuron recordings (50 mW/cm^2^ versus 8 W/cm^2^) and 600-fold less light than QuasAr2 for mouse brain slice recordings (500 mW/cm^2^ versus 300 W/cm^2^). This new GEVI uses a distinct mechanism from other approaches, opening an alternate engineering path to improve sensitivity and speed.

## Introduction

Fluorescent imaging of neuronal activity is ultimately dependent on the number of photons detected. The larger the difference in photons, the easier it is to optically monitor neuronal responses. There are a range of restrictions that limit the number of photons when imaging with Genetically Encoded Voltage Indicators (GEVIs). GEVIs must report multiple states of membrane potential, are limited by expression in the plasma membrane, and need to respond on the millisecond timescale to resolve action potentials. As a result of these restrictions, there are a limited selection of probes that give a fluorescence change large enough to image population signals *in vivo*^1–8^. Confinement to the plasma membrane predisposes probe development to the brightest fluorescent protein (FP) repertoire such as mNeon Green^9^ to image as many photons as possible thereby improving the signal-to-noise ratio (SNR). Red-shifted GEVIs are too dim for imaging populations of cells *in vivo*^10–13^.

The Bongwoori family of probes^14,15^ derived from ArcLight^16^ are GEVIs that fuse a single FP, Super Ecliptic pHluorin A227D (SE227D), to a voltage sensing domain (VSD) and give a large fluorescence change upon depolarization of the plasma membrane. This green FP resides in the cytoplasm at the carboxy-terminus of the membrane-bound GEVI and is pH sensitive^17,18^. Introduction of mutations that inhibit dimerization of the FP reduces the optical signal by more than 70%^19^. This led to the hypothesis that for ArcLight-type probes, dimerization via the FP domain creates a microenvironment that is altered upon membrane depolarizations. Movement of the S4 transmembrane segment in response to voltage relocates the FP altering the interactions with neighboring FPs. In effect, movement of S4 changes the microenvironment of the chromophore most likely due to the A227D mutation of SE227D which introduces a negative charge on the exterior of the β-can^16^.

In this report, we sought to develop a red-shifted GEVI utilizing the potential FP dimerization mechanism. Most red-shifted FPs have been engineered to function as monomers^20^ which make them poor candidates for ArcLight-type probes. A notable exception is FlicR1 that uses the red-shifted FP, cpmApple, which is dim at the resting membrane potential but gets brighter upon depolarization of the plasma membrane^10^. tdTomato is one of the brightest, red-shifted FPs developed^21^ and employs a tandem dimer architecture so that the red-shifted FP effectively functions as a monomer. dTomato is, therefore, an ideal candidate for the development of red-shifted GEVIs. Here, we describe a new red-shifted GEVI, Ilmol (sunset in Korean), that replaces the FP domain of SE227D with dTomato. HEK 293 cells expressing Ilmol are 18 times brighter than cells expressing FlicR1^10^ and exhibit a tenfold larger change in fluorescence per 100 mV depolarization of the plasma membrane. While yielding a smaller fractional change in fluorescence than FlicR1, the increased number of photons detected results in a 3-fold improvement of the SNR. Ilmol expresses well in neurons and is capable of resolving action potentials. Ilmol is also capable of yielding population signals in mouse hippocampal slice making it a viable option for red-shifted voltage imaging despite its small fractional fluorescence change.

## Results

### Linker length affects the optical signal

**Figure 1:**
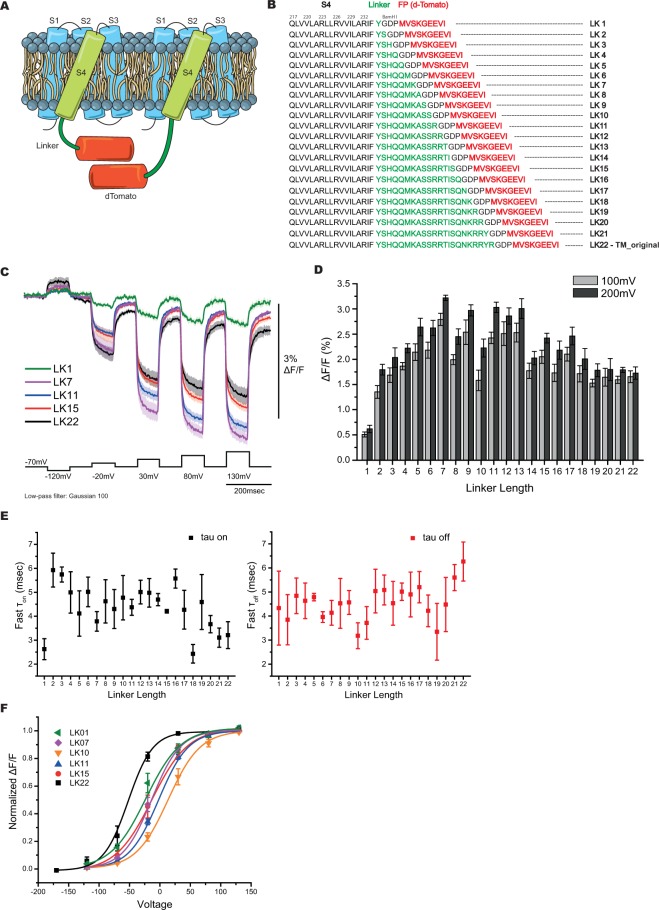
Red-shifted GEVIs with altered linker lengths. (**A**) Schematic presentation of a GEVI with a single dTomato chromophore. The VSD (S1-S4) resides in the plasma membrane with dTomato fused to the carboxy-terminus via a linker segment. (**B**) The amino acid sequence of the varying linker lengths (LK1-LK22) to optimize voltage-dependent signal. The linker segment is in green. dTomato sequence is in red. The GDP sequence between the linker and dTomato is due to a BamHI cloning site. (**C**) Optical traces in response to membrane depolarizations. The average of at least four HEK cells expressing LK1 (green), LK7 (purple), LK11 (blue), LK15 (red), and LK22 (black). The color-coordinated shaded areas are standard errors of the mean. The command voltage pulses are shown below in black. An offline, low-pass Gaussian filter was used for all cells. (**D**) Comparison of the average signal size at 100 mV (grey bars) and 200 mV (black bars) depolarizations of the plasma membrane of all linker lengths tested. (**E**) The fast τ_on_ (black) and fast τ_off_ (red) for each linker tested. (**F**) Boltzmann fit of the normalized fluorescence change in response to membrane potential (n ≥ 4 for all constructs).

To test whether dTomato was a viable FP for GEVI development, dTomato was fused to the VSD of Bongwoori, a modified *Ciona* voltage sensing phosphatase VSD that improved the speed of the optical response of ArcLight^14^. Figure 1A shows a schematic of the red-shifted GEVI design with a single dTomato chromophore fused to the carboxy-terminus enabling a potential intermolecular interaction between neighboring probes. To reduce potential steric hindrance limitations and allow flexibility for the intermolecular dimerization of dTomato, the *Ciona* phosphatase linker sequence consisting of 22 amino acids was used to separate the FP from the VSD (Fig. 1B). This probe, designated LK22, did not give a large fractional change in fluorescence upon depolarization of the plasma membrane when expressed in HEK 293 cells. However, the brightness of this probe (a function of expression level and molecular properties) was similar to that of ArcLight and much brighter than FlicR1 (discussed in the description of the final version of Ilmol) encouraging further development.

Previous reports have shown that altering linker length can affect the signal size of a GEVI^14,22–24^. Twenty-two linker lengths (designated LK1-LK22) were tested by removing one amino acid at a time (Fig. 1B). Figure 1C shows representative traces of the original construct (LK22: −1.7 ± 0.1% ΔF/F/100 mV depolarization), the worst performing construct (LK1: <−1% ΔF/F/100 mV), and two of the better constructs (LK7: −2.8 ± 0.1% ΔF/F/100 mV and LK11: −2.4 ± 0.1% ΔF/F/100 mV) when expressed in HEK 293 cells (Fig. 1D). LK7 and LK11 also had fast time constants (Fig. 1E and Table 1). LK7 had a fast τ_on_ of 4 ± 1 msec which accounted for 71 ± 6% of the total amplitude of fluorescence change per 100 mV depolarization of the plasma membrane. The τ_off_ for LK7 was 4 ± 1 msec accounting for 68 ± 4% of the amplitude. LK11 had a fast τ_on_ of 4 ± 1 msec which accounted for 80 ± 2% of the total amplitude of fluorescence change per 100 mV depolarization of the plasma membrane. The fast τ_off_ for LK11 was also 4 ± 1 msec (72 ± 5% of total amplitude). Like Bongwoori, when the linker sequence was shortened the voltage range of the optical signal shifted to more positive potentials in relation to the original LK22 construct (Fig. 1F). The initial LK22 construct had a V_1/2_ (the membrane potential at half maximum ΔF/F) of −52 ± 13 mV. LK7 had a V_1/2_ of −14 ± 3 mV and LK11 had a V_1/2_ of −2 ± 3 mV. However, some deletions in the linker did cause a smaller shift to more negative potentials (compare LK7 to LK10, Fig. 1F). Given the empirical nature for the voltage ranges of GEVIs^14,24,25^, 200 mV depolarizations were also performed to identify potential probes in need of voltage tuning. All of the constructs showed only a slight increase in the optical signal for 200 mV depolarizations, eliminating the need to further optimize the VSD.

**Table 1:** Physical properties of the 22 linker constructs.

Construct	V_1/2_ (mV)	τ	Weighted τ (msec)	Fast τ (msec)	Slow τ (msec)	% fast τ
LK1	−23 ± 6	on	12 ± 3	3 ± 1	23 ± 6	49 ± 5
		off	15 ± 1	4 ± 2	29 ± 2	55 ± 8
LK2	−2 ± 3	on	21 ± 4	6 ± 1	46 ± 12	61 ± 5
		off	17 ± 6	4 ± 2	40 ± 17	52 ± 13
LK3	−8 ± 2	on	20 ± 5	6 ± 1	45 ± 11	64 ± 5
		off	16 ± 3	5 ± 1	40 ± 10	64 ± 6
LK4	−11 ± 2	on	15 ± 2	5 ± 1	35 ± 5	66 ± 5
		off	11 ± 2	5 ± 1	25 ± 6	65 ± 4
LK5	−5 ± 1	on	18 ± 5	4 ± 1	39 ± 14	58 ± 4
		off	13 ± 1	5 ± 1	35 ± 6	70 ± 6
LK6	−10 ± 1	on	19 ± 4	5 ± 1	52 ± 8	69 ± 3
		off	13 ± 2	4 ± 1	36 ± 8	71 ± 3
LK7	−14 ± 3	on	11 ± 1	4 ± 1	30 ± 4	71 ± 6
		off	12 ± 2	4 ± 1	28 ± 3	68 ± 4
LK8	−4 ± 4	on	21 ± 6	5 ± 1	50 ± 18	57 ± 6
		off	15 ± 5	5 ± 2	34 ± 13	61 ± 3
LK9	−11 ± 1	on	15 ± 3	4 ± 1	38 ± 10	63 ± 9
		off	13 ± 1	5 ± 1	26 ± 5	60 ± 6
LK10	14 ± 3	on	16 ± 3	5 ± 1	32 ± 9	53 ± 8
		off	15 ± 3	3 ± 1	26 ± 5	62 ± 4
LK11	−2 ± 3	on	11 ± 3	4 ± 1	38 ± 10	80 ± 2
		off	10 ± 2	4 ± 1	27 ± 6	72 ± 5
LK12	−12 ± 7	on	14 ± 2	5 ± 1	38 ± 7	71 ± 3
		off	17 ± 5	5 ± 1	41 ± 20	59 ± 8
LK13	−5 ± 5	on	14 ± 2	5 ± 1	31 ± 3	65 ± 3
		off	13 ± 3	5 ± 1	25 ± 4	61 ± 5
LK14	−13 ± 3	on	15 ± 5	5 ± 1	43 ± 17	74 ± 5
		off	11 ± 2	5 ± 1	26 ± 5	69 ± 4
LK15	−13 ± 6	on	12 ± 2	4 ± 1	36 ± 4	75 ± 3
		off	10 ± 2	5 ± 1	23 ± 2	73 ± 9
LK16	1 ± 6	on	18 ± 5	6 ± 1	62 ± 10	78 ± 5
		off	10 ± 2	5 ± 1	32 ± 13	70 ± 9
LK17	−10 ± 5	on	15 ± 5	4 ± 1	32 ± 9	70 ± 3
		off	11 ± 2	5 ± 1	34 ± 5	74 ± 5
LK18	−12 ± 4	on	9 ± 1	2 ± 1	27 ± 5	71 ± 4
		off	8 ± 2	4 ± 1	25 ± 5	81 ± 5
LK19	−29 ± 6	on	17 ± 7	5 ± 2	39 ± 16	62 ± 5
		off	11 ± 3	3 ± 2	19 ± 3	52 ± 8
LK20	−28 ± 5	on	11 ± 2	4 ± 1	40 ± 9	78 ± 4
		off	11 ± 3	4 ± 2	33 ± 14	66 ± 11
LK21	−32 ± 9	on	10 ± 2	3 ± 1	27 ± 4	71 ± 2
		off	16 ± 3	6 ± 1	56 ± 18	76 ± 4
LK22	−52 ± 13	on	16 ± 4	3 ± 1	36 ± 5	62 ± 5
		off	15 ± 4	6 ± 1	29 ± 7	63 ± 9

### The charge composition of the linker also affects the optical signal

**Figure 2:**
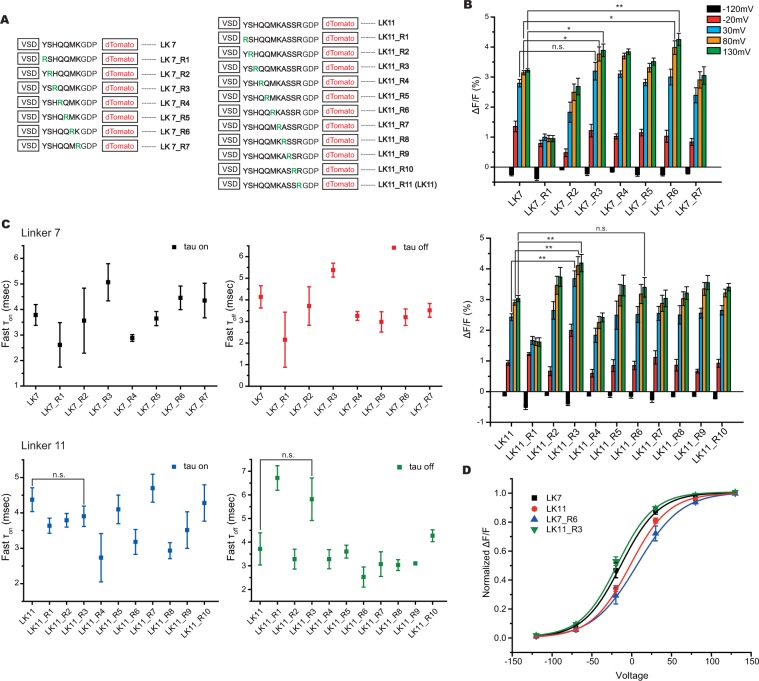
Introduction of positive charges into the linker segment improved the voltage-dependent optical signal. (**A**) List of constructs showing the position of the positively charged amino acid, arginine, in the LK7 construct (left) and the LK11 construct (right). The arginine mutation is shown in green. (**B**) Average optical responses to hyperpolarization of the plasma membrane to −120 mV (black), depolarization of the plasma membrane to −20 mV (red), depolarization to 30 mV (blue), depolarization to 80 mV (orange), and depolarization to 130 mV (green). (**C**). Fast time components of all the constructs presented in A. (**D**) Boltzmann fit of the normalized fluorescence change for the two best constructs in B. LK7 and LK11 are replotted for ease of comparison. Asterisks in B indicate statistically significant differences between means compared (n.s. no significant difference, *p < 0.05, **p < 0.01). (n ≥ 4 for all constructs).

Introducing positively charged amino acids in the linker segment improved the Bongwoori family of probes resulting in two new GEVIs, Bongwoori-Pos6 and Bongwoori-R3^15^. We performed a similar analysis on the dTomato GEVIs. Positive charges were introduced at every position in the linker separating the VSD from the FP for both the LK7 and LK11 constructs (Fig. 2A). Introduction of an arginine residue at the third position in the linker for the LK11 construct (LK11_R3) improved the optical signal for the 100 mV voltage step (LK11: −2.4 ± 0.1% versus LK11_R3: −3.7 ± 0.3%, Fig. 2B and Table 2). Interestingly, the same mutation in the LK7 linker did not significantly alter the signal size for the 100 mV pulse but did improve the optical signal for the 150 mV and 200 mV depolarization steps. This may be indicative of the linker length affecting the orientation of the FP domain of the GEVI. Another example is the R6 position. LK7_R6 increased the 200 mV depolarization signal 33% but had very little effect on the LK11_R6 signal. The R6 position in the LK7 construct may contribute more to the orientation of the FP domain than it does in the 11 amino acid linker sequence. The charge composition also had an effect on the voltage sensitivities of the probes. The arginine at position 6 in the LK7 construct shifted the V_1/2_ to more positive potentials while an arginine at position 3 in the LK11 construct shifted to more negative potentials.

**Table 2:** Physical properties of the arginine scan series of constructs.

Construct	State	Weighted τ (msec)	Fast τ (msec)	Slow τ (msec)	% fast
LK7_R1	on	8 ± 2	3 ± 1	17 ± 5	58 ± 15
	off	15 ± 2	2 ± 2	24 ± 5	37 ± 8
LK7_R2	on	10 ± 3	4 ± 2	22 ± 5	64 ± 7
	off	10 ± 3	4 ± 1	32 ± 10	73 ± 6
LK7_R3	on	14 ± 1	5 ± 1	24 ± 1	56 ± 3
	off	12 ± 1	5 ± 1	19 ± 2	52 ± 7
LK7_R4	on	8 ± 1	3 ± 1	23 ± 2	72 ± 3
	off	8 ± 1	3 ± 1	28 ± 5	81 ± 3
LK7_R5	on	10 ± 1	4 ± 1	29 ± 4	74 ± 2
	off	10 ± 2	3 ± 1	30 ± 10	68 ± 5
LK7_R6	on	12 ± 2	4 ± 1	41 ± 5	79 ± 6
	off	9 ± 2	3 ± 1	24 ± 6	68 ± 5
LK7_R7	on	11 ± 2	4 ± 1	30 ± 7	71 ± 4
	off	10 ± 1	4 ± 1	25 ± 4	70 ± 5
LK11_R1	on	11 ± 1	4 ± 1	32 ± 6	73 ± 4
	off	15 ± 2	7 ± 1	29 ± 7	57 ± 10
LK11_R2	on	9 ± 1	4 ± 1	30 ± 3	79 ± 3
	off	6 ± 1	3 ± 1	19 ± 3	80 ± 4
LK11_R3	on	13 ± 1	4 ± 1	30 ± 3	66 ± 4
	off	15 ± 1	6 ± 1	26 ± 5	54 ± 8
LK11_R4	on	10 ± 1	3 ± 1	23 ± 5	60 ± 10
	off	11 ± 1	3 ± 1	21 ± 3	54 ± 11
LK11_R5	on	11 ± 1	4 ± 1	29 ± 4	72 ± 4
	off	12 ± 2	4 ± 1	33 ± 6	71 ± 3
LK11_R6	on	8 ± 2	3 ± 1	23 ± 5	75 ± 4
	off	7 ± 1	3 ± 1	19 ± 3	73 ± 7
LK11_R7	on	14 ± 2	5 ± 1	40 ± 8	72 ± 6
	off	10 ± 2	3 ± 1	28 ± 7	73 ± 4
LK11_R8	on	7 ± 1	3 ± 1	22 ± 2	78 ± 2
	off	8 ± 1	3 ± 1	26 ± 3	80 ± 3
LK11_R9	on	10 ± 1	4 ± 1	30 ± 3	76 ± 3
	off	7 ± 1	3 ± 1	19 ± 3	76 ± 2
LK11_R10	on	11 ± 3	4 ± 1	28 ± 7	71 ± 3
	off	11 ± 2	4 ± 1	29 ± 2	73 ± 5
LK7_R6_K159R	on	11 ± 2	4 ± 1	29 ± 3	71 ± 5
	off	9 ± 2	3 ± 1	18 ± 2	63 ± 5
LK11_R3_K159R	on	10 ± 1	4 ± 1	26 ± 4	72 ± 5
	off	11 ± 1	6 ± 1	27 ± 4	74 ± 3

The time constant, τ, represents the time it takes for the fluorescence to reach 63% of the maximum fractional change. The τ_on_ therefore can provide a simple approximation of the signal size expected from an action potential. For instance, a GEVI with a τ_on_ of 10 msec will reach about 18% of the maximal signal in 2 msec while a GEVI with a τ_on_ of 1 msec would reach 86% of its maximum signal (as calculated from a single exponential decay – Table 3). The τ_off_, conversely, estimates the length of time to return to 37% of the baseline fluorescence providing a rough estimate of the firing frequency of action potentials that can be optically resolved. The speed of the optical responses for the altered linker compositions varied between 2.5 to 5.0 msec for the τ_ons_ and between 2.5 and 7.0 msec for the τ_offs_ (Fig. 2C). These speeds are a slight improvement of the Bongwoori family of probes warranting further characterization in primary neuronal cultures.

**Table 3:** Percentage of maximum fluorescence change during a 2 msec action potential.

τ_on_ (msec)	% of maximum signal at 2 msec
1	86
2	63
3	49
4	39
5	33
6	28
7	25
8	22
9	20
10	18

Approximating an action potential as a 100 mV depolarization that lasts for 2 msec enables the estimation of the optical signal as a function of τ_on_ when fit to a single exponential decay (F_t_ = F_max_(1 − *e* ^−t/τ^) where F max is 100% and t is 2 msec).

### Introducing charged residues to the exterior of dTomato

**Figure 3:**
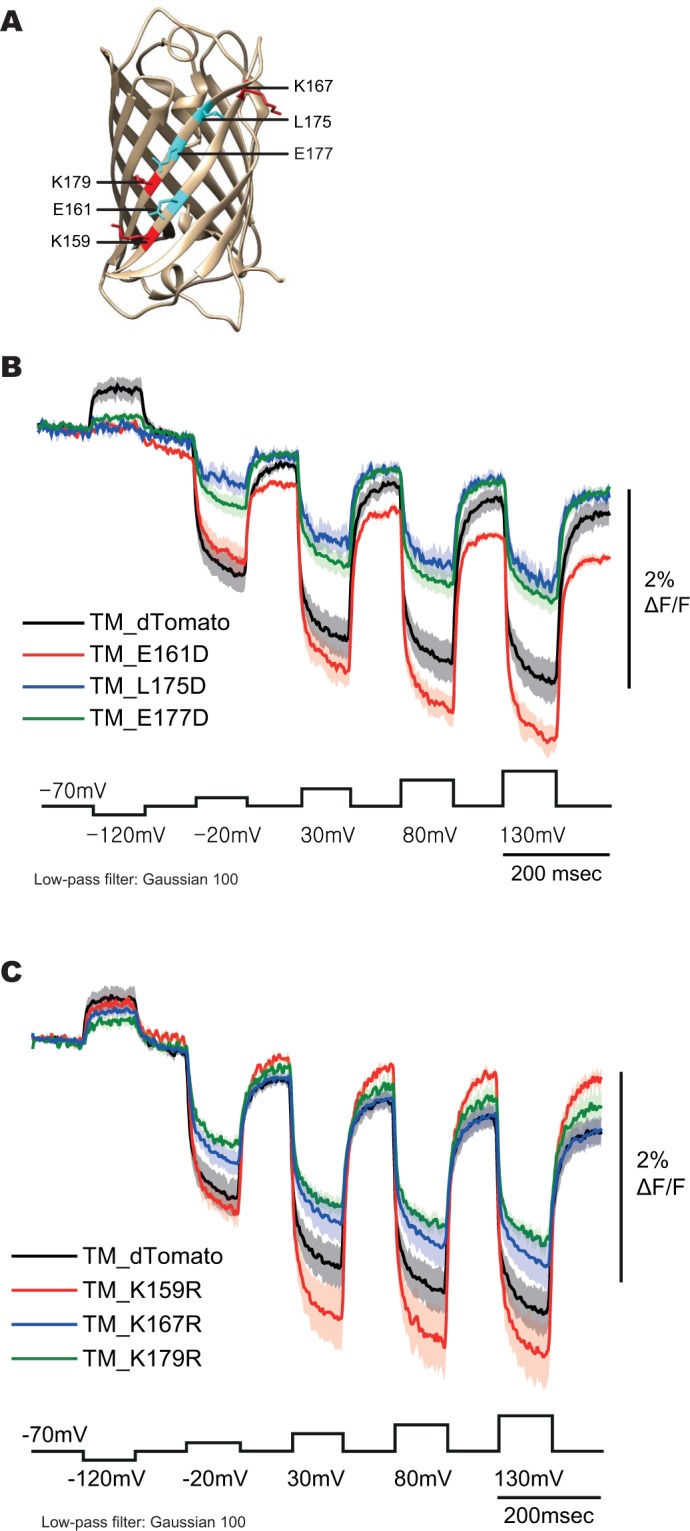
Aspartic acid and arginine scanning mutagenesis of dTomato. (**A**) Crystal structure of dsRed^28^. (**B**) Optical traces of HEK cells expressing the aspartic acid scan mutants. Command voltage pulses are in black. (**C**) Optical traces of HEK cells expressing the arginine scan mutants. Command voltage pulses are in black (n ≥ 4 for all constructs).

The serendipitous A227D mutation to the FP in ArcLight improved the voltage-dependent optical signal 15-fold^16^. The mechanism behind this signal increase is not well understood but may have something to do with the pH-sensitivity of SE227D^19,26,27^. The external, negative charge enables conformational changes in the VSD to alter the microenvironment of the GEVI^28^. Since the pKa of dTomato is much lower than SE227D^16,20^, the exterior residues of the β-can near the dimer interface of the FP (Fig. 3A) were replaced with both the negatively charged aspartic acid residue or the positively charged arginine residue (all mutations are listed in Supplementary Table 1), in an attempt to improve the dynamic range of the voltage response of the optical signal. The LK22 construct was again used to limit potential steric hindrance limitations induced by shorter linker lengths.

Most mutations failed to fluoresce when expressed in HEK 293 cells (Supplementary Table 1). The K159R (amino acid number based on dTomato sequence) showed a slight improvement of the optical signal to 2.5% ΔF/F at 100 mV depolarization (Fig. 3B,C). The E161D construct was not used for further development due to the apparent increased bleaching of the FP exhibited by the decay in the baseline (Fig. 3B).

### An improved, red-shifted GEVI, Ilmol

**Figure 4:**
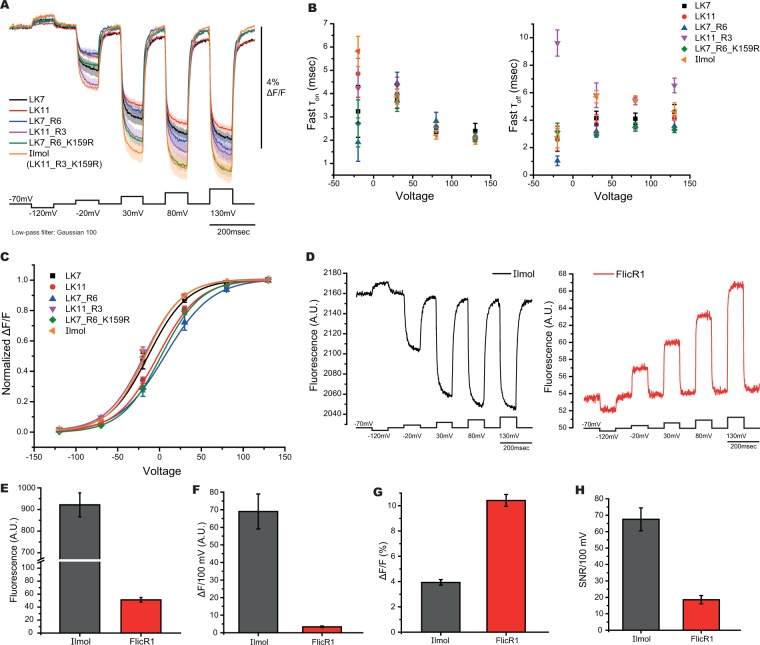
Optical properties of Ilmol. (**A**) Optimizing multiple parameters slightly improves signal size. Traces from two linker lengths, LK7 and LK11, were compared to the optimized linker charge composition, LK7_R6 and LK11_R3, and FP mutations, LK7_R6_K159R and LK11_R3_K159R (Ilmol). (**B**) Time constants of constructs in A. (**C**) Voltage range of constructs in A. (**D**) Comparison of Ilmol to FlicR1. Representative traces of Ilmol (left) or FlicR1 (right) in response to voltage when expressed in HEK 293 cells. The voltage protocol was the same for both constructs (average of 16 trials). (**E**) Average brightness for Ilmol and FlicR1 (n > 60 for both constructs in HEK 293 cells). (**F**) Average change in fluorescence for Ilmol and FlicR1 per 100 mV depolarization of the plasma membrane when expressed in HEK 293 cells. (**G**) Average fractional fluorescence change per 100 mV. (**H**) SNR for 100 mV depolarization of the plasma membrane (n ≥ 4 for all constructs).

Combining the optimized parameters resulted in a construct that yielded a 4.2 ± 0.3% ΔF/F/100 mV depolarization step of the plasma membrane (Fig. 4A, Table 2). This new GEVI, Ilmol, uses the 11 amino acid linker length with an arginine residue at position three in the linker segment with the K159R mutation to dTomato. Ilmol is faster than Bongwoori with the τ_on_ under three msec for a 100 mV depolarization of the plasma membrane and the τ_off_ being close to six msec upon repolarization to the resting potential (Fig. 4B and Table 2). The V_1/2_ of Ilmol is around −20 mV (Fig. 4C and Table 2).

**Figure 5:**
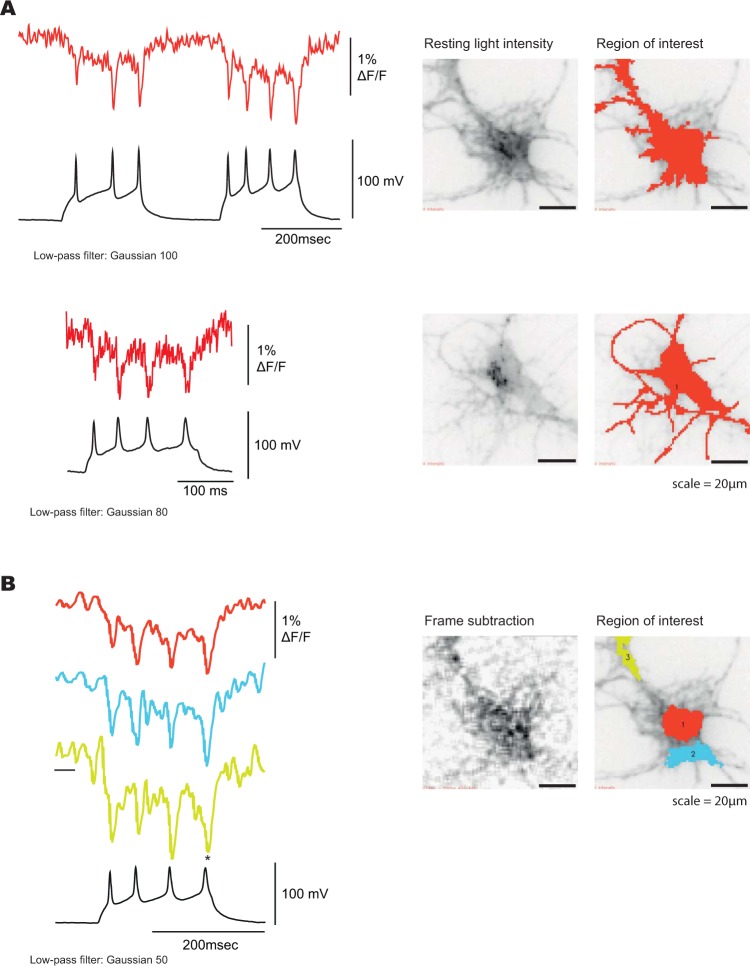
Optical signal from Ilmol in response to action potentials. (**A**) Hippocampal neurons expressing Ilmol were imaged under current clamp. Top trace is a higher quality signal from a single trial recording at 1000 hertz yielding a 1.5% signal/action potential. Bottom trace is a lower quality signal giving a 1% signal per action potential. Corresponding neurons are shown on the right. Resting fluorescence is the grey scale image. Regions of interest generating traces on the left are shown in red. (**B**) Optical traces of Ilmol from different regions of a neuron (soma: red, processes: blue and yellow). The corresponding region of interest is shown on the right. Image on the left shows the changes in fluorescence during action potential. A frame subtraction image was generated by subtracting the average fluorescence of the three frames during the action potential (denoted by an asterisk) from the average fluorescence of 20 frames before the onset of action potentials (denoted by black bar below the yellow trace).

Ilmol is about 18 times brighter than FlicR1 (Fig. 4D,E, excitation wavelength 561 nm, intensity 50 mW/cm^2^). FlicR1 has the advantages of being faster and possessing a larger fractional change of the fluorescence in response to voltage (Fig. 4D,F). Ilmol, however, gives a ten-fold larger change in fluorescence per 100 mV depolarization step resulting in an improved signal-to-noise response (Fig. 4G). The photostability of Ilmol is comparable to FlicR1 (Supplementary Fig. 1). Ilmol expressed well in mouse hippocampal neurons resolving action potentials in single trials (Fig. 5, Supplementary Fig. 3).

### Population recordings from mouse hippocampal acute brain slice

**Figure 6:**
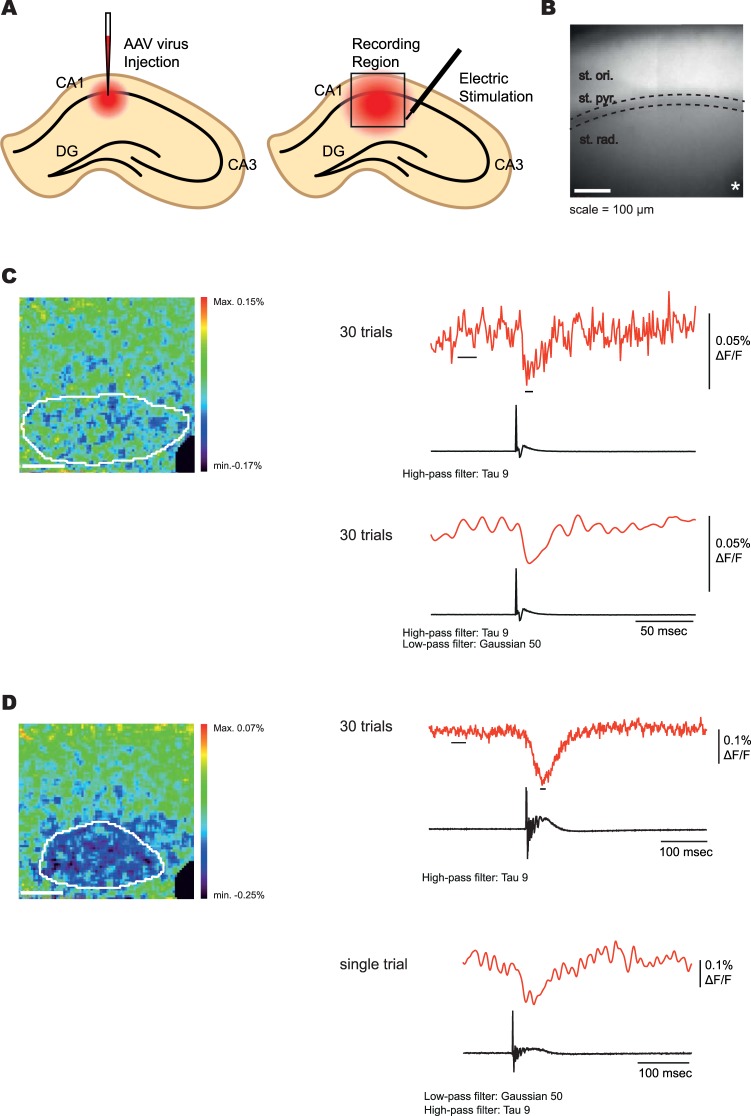
Population recording from mouse brain slice with Ilmol. (**A**) Schematic of the injection site into the CA1 region of the mouse brain. (**B**) The resting light image of an acute brain slice expressing Ilmol. The pyramidal cell layer resides inside the dotted lines. The asterisk denotes the site of the stimulating electrode. An electrode recording the field potential was placed in the pyramidal cell layer. (**C**) Heat map of neuronal activity. The image is the same as in B. The fluorescence trace (red) is from all of the pixels inside the white circle in the heat map. The heat map was generated by subtracting the average of five frames post stimulation (indicated by black dash under the fluorescence trace) from the average of 20 frames pre-stimulation (represented by the bar below the fluorescence trace). The field potential recording is shown in the black trace below to indicate the onset of stimulation. The stimulation electrode was masked (black pixels in lower right hand corner). (**D**) Effect of bicuculline on neuronal activity. The heat map was generated in a similar fashion as in C. Fluorescence traces again correspond to the pixels inside the white circle. Top fluorescence trace is the average of 30 trials. Bottom fluorescence trace is of a single trial. Offline filtering is as indicated below each trace. All recordings were done at 1000 frames per second. Light intensity was 500 mW/cm^2^.

To investigate the ability of Ilmol to report neuronal activity in brain slice, an adeno associated (AAV) virus of Ilmol was constructed using the hSynapsin promoter to drive expression. The Ilmol AAV was injected into the CA1 region of the mouse hippocampus (Fig. 6A,B). Two weeks post injection, acute brain slices were imaged during field stimulation of the Schaeffer collateral axons. An optical signal was detected in acute brain slices from two of the three mice injected (Fig. 6C,D, and Supplementary Fig. 4). Brain slices from the third mouse failed to fluoresce probably due to a problem during the injection of the virus. Figure 6C shows the average optical signal from 30 trials. The heat map was generated by subtracting frames post stimulation from frames prior to stimulation. All pixels inside the white circle were used to generate the optical trace. There was no need to restrict the region of interest to only those pixels demonstrating the largest fluorescence change. Upon the addition of Bicuculline, Ilmol was able to report post synaptic activity in a single trial (Fig. 6D). Ilmol was also able to report neuronal activity during a paired pulse stimulation protocol (Supplementary Fig. 4).

## Discussion

Our improved understanding of the mechanism enabling membrane potential to mediate a fluorescence response for ArcLight-type probes has led to the development of a very bright, red-shifted GEVI. Super Ecliptic pHluorin is an environmentally sensitive fluorescent probe^17^ that when fused to a VSD, gives a small, voltage-dependent, optical signal^22^. A fortuitous mutation that introduced a negative charge on the outside of the β-can structure of Super Ecliptic pHluorin resulted in large signals up to 40% ΔF/F/100 mV^22^. During the development of the GEVI, Pado, the introduction of mutations that favor the monomeric form of GFP^29^ reduced the voltage-dependent signal to under 1% ΔF/F/100 mV^19^. This need for an intermolecular association of the FP encouraged the use of dTomato resulting in the brightest red-shifted GEVI developed to date.

Like other GEVIs, the linker length and amino acid composition affected the voltage-dependent signal^7,14,15,22–24^. Unfortunately, our efforts to put charges on the outside of the β-can of dTomato did not result in a substantial increase in the voltage-dependent, optical signal. The wrong positions were chosen and/or the pH sensitivity of dTomato needs to be optimized. Regardless, this approach opens a new frontier in GEVI development. Using the dimerization of the FP to create a microenvironment affecting the chromophore could result in the generation of new ArcLight-type GEVIs with a pKa outside physiological ranges. For instance, the introduction of charged amino acids could create an appropriate environment that would affect the chromophore upon movement of S4 in response to voltage similar to that found for GCaMP^26^. Such an approach highlights the need for rational GEVI development to complement recently developed high throughput approaches^13,30^. With such a large parameter space, high throughput approaches are fundamental to GEVI development. However, only a rational approach based on a mechanism hypothesis would have resulted in a red-shifted dimer probe. Hopefully, armed with this new starting position, directed evolution will further improve Ilmol.

Voltage imaging of neuronal activity is influenced by a broad spectrum of parameters. The speed of the GEVI will dictate the size of the optical signal for an action potential and the firing frequency that probe can resolve. The brightness of the probe (F) will dictate the light intensity required for imaging and the potential number of photons that can be varied upon voltage changes. The voltage range of the probe will dictate the types of neuronal activity that can be monitored. Photostability and expression patterns of the probe also play a role. With this myriad of parameters contributing to the optical output, it is difficult to state with certainty which probe is best. The experimenter should instead ask what GEVI has the most optimized characteristics for the recording being attempted. Here, we report the generation of a very bright, red-shifted GEVI capable of yielding population signals in acute slice recordings which should provide a good complement to the toolbox of GEVIs currently available to the neuroscience community.

## Material and Methods

### Plasmid design and construction

The TM_dTomato probe was made by modifying the Triple mutant construct reported previously^14^. A BamHI restriction site was added downstream of the VSD to facilitate cloning of different linker lengths. A single copy variant of tdTomato was synthesized with a 5′ BamHI site and a 3′ stop codon followed by a XhoI site (Integrated DNA Technology, USA)^21^.

Single-step PCR was used to systematically decrease the length of the linker between the VSD and the FP. Final constructs were ligated into pcDNA3.1/Hygro (+) backbone vector (Invitrogen, USA).

Arginine scanning mutagenesis of the linker region was done by a two-step PCR process using LK7 and LK11 as template DNA. Primers were designed to introduce arginine mutations in each amino acid site of the linker. Constructs tested in the arginine scanning mutagenesis of dTomato were generated by two-step PCR using TM_dTomato as template DNA.

DNA of FlicR1 was obtained from Addgene (#74142; Addgene, USA) and cloned into pcDNA3.1/Hygro (+) backbone vector (Invitrogen, USA). All DNA constructs were verified by DNA sequencing (Cosmogenetech, Republic of Korea).

### Cell culture and transfection

HEK293 cells were maintained in Dulbecco’s modified Eagle medium (high glucose DMEM; Gibco, USA) supplemented with 10% fetal bovine serum (FBS; Gibco, USA). HEK293 cells were seeded on to #0 coverslips (Ted Pella Inc., USA) coated with poly-L-lysine (Sigma-Aldrich, USA). Expression of constructs in HEK 293 cells and hippocampal neuronal cultures was achieved using Lipofectamine 2000 (Invitrogen, USA). Transfection was done according to the manufacturer’s protocol. To optimize the expression level of the probes, FlicR1 was recorded 48 hours after transfection, whereas other probes were recorded after 24 hours^10^.

Hippocampal neurons were obtained according to an approved animal experiment protocol by the Institutional Animal Care and Use Committee at KIST (animal protocol 2016-082). Primary hippocampal neurons were obtained from C57BL6/F mice (Koatech, Republic of Korea) on embryonic day 18 as described previously^31^. Dissociated neurons were obtained by digesting hippocampi with 0.05% trypsin-EDTA solution (Gibco, USA) for 10 minutes at 37 °C. Neurons were then dissociated by mechanical trituration through Pasteur pipettes (Hilgenberg, Germany). Dissociated neurons were plated onto #0 coverslips (Ted Pella Inc., USA) coated with poly-D-lysine (Sigma-Aldrich, USA) at 5 × 10^4^ cells/ml density. After seeding, the neurons were cultured at 37 °C in Neurobasal medium (Gibco, USA) supplemented with B-27 (Gibco by Life Tech, USA), 0.5 mM Glutamax-I (Gibco, USA), L-glutamic acid (Sigma, USA), and 0.5% FBS. Transient transfection for neurons was done on 5–7 days *in vitro* (DIV). Neurons were recorded at DIV 8–10.

### Viral injection and slice preparation

Virus injection and slice preparation was done according to an approved animal experiment protocol by the Institutional Animal Care and Use Committee at KIST (animal protocol 2017–031). C57BL/6 N male mice were used in the experiment. Mice over 4 weeks old were first anesthetized by 1.5%~3% isoflurane. Then associated adeno virus (AAV) AAV2/1-hSyn-Ilmol (titer 2.8 × 10^12^ GC/ml; KIST Virus Facility, Republic of Korea) was injected into hippocampus CA1 as previously described^32^. The recording was done at least 2 weeks after the injection.

Acute coronal brain slices (≈1.7 mm posterior to bregma) were made in 300 µm thickness. The mice were put into deep anesthesia by halothane (Sigma, USA) before euthanasia. Isolated brain was sliced by VT-1200 Vibratome (Leica, Germany) in a cold high-sucrose artificial cerebrospinal fluid (ACSF) solution. The solution contains 75 mM Sucrose, 25 mM NaHCO_3_, 2.5 mM KCl, 0.5 mM CaCl_2_, 7 mM MgCl_2_, 1.25 mM NaH_2_PO_4_, 87 mM NaCl and 25 mM D-glucose (pH 7.4), by gassing with 95% O_2_/5% CO_2_. Slices were then placed in an interface chamber filled with ACSF, incubated at 36 °C for at least 1 hour to allow recovery. The solution contains 125 mM NaCl, 2.5 mM KCl, 1.25 mM NaH_2_PO_4_, 25 mM NaHCO_3_, 25 mM D-glucose, 2.5 mM CaCl_2_, 1 mM MgCl_2_, and 1 mM ascorbic acid oxygenated, with 95% O_2_/5% CO_2_.

### Patch clamp (electrophysiology)

Transfected HEK293 cells were patched at 33 °C and the chamber was perfused with bath solution containing 150 mM NaCl, 4 mM KCl, 2 mM CaCl_2_, 1 mM MgCl_2_, 5 mM D-Glucose, and 5 mM HEPES (pH 7.4). 1.5/0.84 mm Glass capillary tube (World Precision Instruments, USA) was pulled by P-97 micropipette puller (Sutter Instruments, USA) to make patch pipette with 3–5 MΩ resistance. The pipette solution contained 120 mM K-aspartate, 4 mM NaCl, 4 mM MgCl_2_, 1 mM CaCl_2_, 10 mM EGTA, 3 mM Na2ATP, and 5 mM HEPES (pH 7). In transfected HEK293 cells, whole-cell voltage clamp was done and current clamp was done in transfected hippocampal neurons using an EPC10 amplifier (HEKA, USA) with a −70 mV holding potential.

Mouse brain slice was also recorded at 33 °C while the chamber was perfused with ASCF. For bicuculline recordings, 80 μM bicuculline was added to the perfusion solution. During the imaging of the slice, 50 µs single square pulses were applied every 20 seconds to Schaffer collateral of the hippocampal CA1 region through a bipolar tungsten electrode (30201; FHC, USA), connected to an isolator (DS3; Digitimer Ltd., UK). The field potential was simultaneously monitored from the CA1 stratum pyramidale layer by an ACSF-filled glass capillary pipette. To diminish the baseline drift of the voltage, 0.1 Hz high-pass filter was applied during the recording. The stimulus timing trigger and recording were done using Multiclamp 700B amplifier (Molecular devices, USA).

### Fluorescence imaging

An IX71 microscope with a 60 × 1.35 numerical aperture oil-immersion lens (Olympus, Japan) was used to image fluorescence during patch-clamp experiments. The light source was a 75 W Xenon arc lamp (Osram, Germany) placed in a lamp housing (Cairn, UK). The excitation filter was FF01-561/14 (Semrock, USA), the emission filter was FF01–609/54 (Semrock, USA), and the dichroic mirror was Di02-R561 (Semrock, USA). The objective C-mount image was demagnified by an Optem zoom system A45699 (Qioptiq LINOS, USA) projected onto 80 × 80 e2v CCD39 chip of NeuroCCD-SM 80 camera (RedShirtImaging, USA). Image recording frame rate was 1000 frames/sec. The entire imaging devices were mounted on anti-vibration table (Daeil systems, Republic of Korea). The light source, amplifier, mechanical shutter, and manipulator were mounted on a separate table and did not touch the anti-vibration table.

Photobleaching experiment of HEK 293 cells expressing Ilmol and FlicR was done using same light source, filter, and camera. The excitation light intensity measured at the specimen plane was 50 mW/cm^2^. 40 sec light-on and 20 sec light-off state constituted one trial and each trial was repeated to bleach the cells on the coverslip. The patch chamber was maintained in the same condition as patch clamp experiment. Image recording frame rate for bleaching experiment was 40 frames/sec during 40 sec light-on state. Fluorescence intensities were spatially averaged from the whole cell area and plotted as a function of emitted fluorescence versus cumulative excitation time.

The confocal images were acquired by a A1R confocal microscope (Nikon, Japan). 561 nm laser was used for excitation and 595/50 nm bandpass filter was used for emission. The samples for confocal imaging were fixed with 4% paraformaldehyde solution in phosphate buffered saline adjusted at pH 7.4 and mounted with antifade mounting medium with DAPI (H-1500; Vector Laboratories, USA).

For slice recording, an upright epifluorescence microscope (Slicescope; Scientifica, UK) with a 10 × 0.3 numerical aperture water immersion objective lens (Olympus, Japan) was used. The light source was UHP-T-LED-White-High-CRI (Prizmatix, Israel). The excitation filter was FF01–562/40, the emission filter was FF02–641/75, and the dichroic mirror was FF593-Di03. The camera used to image optical response is same as the one used in cultured cells.

### Data acquisition and analysis

Optical signal recordings were analyzed using Neuroplex software (RedshirtImaging, USA), Excel (Microsoft, USA), and Origin8.6 (Origin Labs, USA). The fluorescent traces for constructs expressed in HEK293 cells were averages of 16 trials and traces from neurons were from a single trial. The offline low-pass temporal filtering used is indicated in the figure legends. For the kinetics, the optical traces were fitted to a double exponential decay,

$${\rm{y}}={{\rm{y}}}_{0}+{\rm{A}}1{{\rm{e}}}^{-({\rm{t}}-t0)/\tau 1}+{\rm{A}}2{{\rm{e}}}^{-({\rm{t}}-{\rm{t}}0)/{\rm{\tau }}2}$$

where t is time in milliseconds, or a single exponential decay,

$${\rm{y}}={{\rm{y}}}_{0}+{\rm{A}}1{{\rm{e}}}^{-({\rm{t}}-{\rm{t}}0)/{\rm{\tau }}1},$$

where t is time in milliseconds.

To compare the optical responses that were better fitted to a single exponential decay to those better fitted to a double exponential decay, a weighted τ was calculated as the sum of τ1 multiplied by the relative amplitude, A1, plus τ2 multiplied by the relative amplitude, A2, as defined by the following equation:

$${\rm{\tau }}\,{\rm{weighted}}={\rm{\tau }}1[{\rm{A}}1/({\rm{A}}1+{\rm{A}}2)]+{\rm{\tau }}2\,[{\rm{A}}2/({\rm{A}}1+{\rm{A}}2)].$$

The voltage sensitivity was determined by initially fitting individual cell responses to the Boltzmann equation:

$${\rm{y}}=({\rm{A}}1-{\rm{A}}2)/[1+{{\rm{e}}}^{({\rm{x}}-{\rm{x}}0)/{\rm{dx}}}]+{\rm{A}}2$$

where y is −ΔF/F, and x is membrane potential in mV. A1 is the minimum value, and A2 is the maximum value. x0 is the membrane potential in mV at half maximal ΔF/F, and dx is the slope at x0. All traces were then normalized such that A1 = 0 and A2 = 1. The trials for each construct were then averaged and refitted.

Signal-to-Noise Ratios (SNRs) of Ilmol and FlicR1 were calculated by dividing average ΔF/F of last 20 frames at 100 mV depolarizing stimulation by noise value, which was determined by the standard deviation of the first 50 frames before any electrical stimulation.

Prior to statistical analyses to compare means from different groups, normality of the data was determined by Shapiro-Wilk test. The means from two different groups following normal distribution were compared by a two tailed Student’s t-test.

## Electronic supplementary material

Supplementary information

## Data Availability

The datasets generated during and/or analyzed during the current study are available from the corresponding author on reasonable request.
