# Supplementary material for: A dimeric fluorescent protein yields a bright, red-shifted GEVI capable of population signals in brain slice
Source: Sci Rep. 2018 Oct 12;8:15199. doi: 10.1038/s41598-018-33297-y (PMC6185910; doi:10.1038/s41598-018-33297-y)
Supplement: Supplementary file 1 — Supplementary information [file 41598_2018_33297_MOESM1_ESM.pdf]

A dimeric fluorescent protein yields a bright, red-shifted GEVI capable of population signals in brain slice

Bumjun Yi[1,2], Bok Eum Kang[1,2], Sungmoo Lee[1,3], Sophie Braubach[1], and Bradley J. Baker[1,2]

1. The Center for Functional Connectomics, Korea Institute of Science and Technology, Seoul, Republic of Korea
2. Division of Bio-Medical Science and Technology, KIST School, Korea University of Science and Technology(UST), Seoul, Republic of Korea
3. Department of Transdisciplinary Studies, Graduate school of Convergence Science and Technology, Seoul National University, Suwon, Republic of Korea

Correspondence to bradley.baker19@gmail.com

	Construct	Fluorescence	Voltage Signal
**Arg Scan**	A146R	none	
	T148R	none	
	Y152R	none	
	**K159R**	**yes**	**yes**
	E161R	none	
	H163R	none	
	A165R	none	
	**K167R**	**yes**	**yes**
	H173R	**yes**	no
	L175R	none	
	E177R	none	
	**K179R**	**yes**	**yes**
	Y193R	none	
	Y195R	none	
**Asp Scan**	A146D	none	
	T148D	none	
	R150D	none	
	Y152D	none	
	R154D	**yes**	no
	K159D	none	
	**E161D**	**yes**	**yes**
	H163D	none	
	A165D	none	
	K167D	none	
	H173D	none	
	**L175D**	**yes**	**yes**
	**E177D**	**yes**	**yes**
	K179D	none	
	Y193D	none	
	R221D	none	

Supplementary Table 1. List of mutations to dTomato. Amino acid numbers refer to the position in dTomato. The Probes were tested in HEK 293 cells, n≥4 for all constructs. Excitation was 560 nm. The emitted light was through a band pass filter ranging from 580 nm to 640 nm. The light intensity was 50 mW/cm^2.

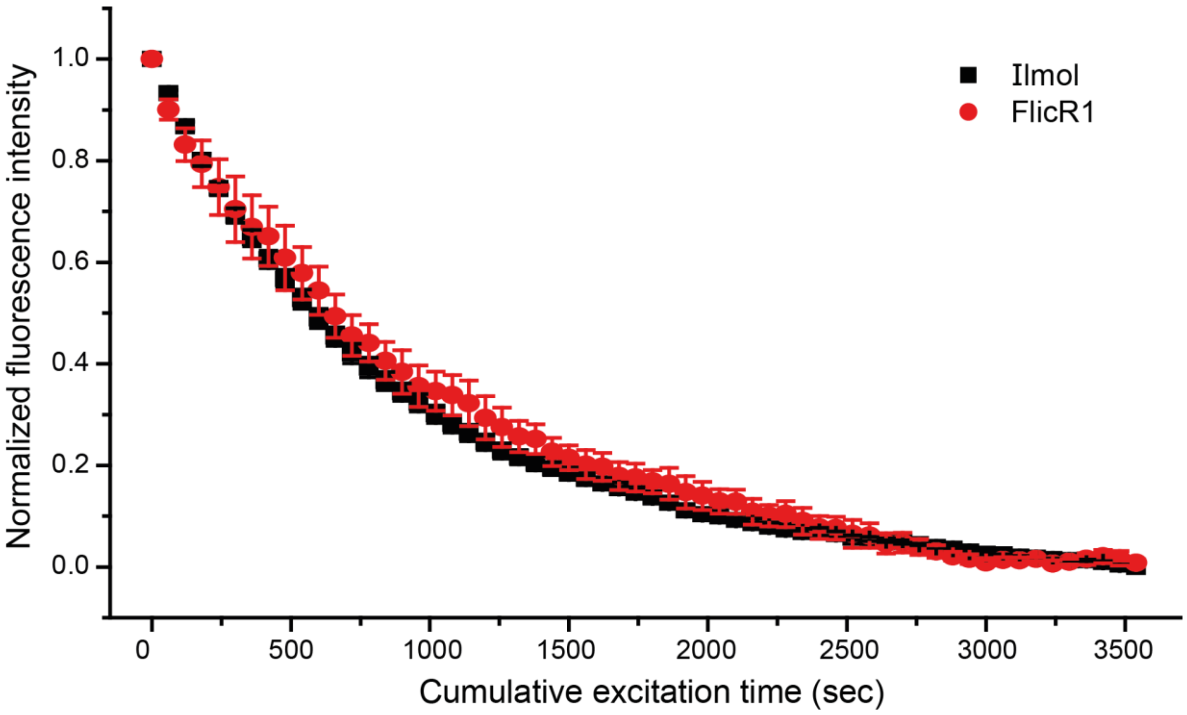

(n≥4 for all constructs)

Supplementary Figure 1. Comparison of bleaching rates for Ilmol and FlicR1. Experiment condition listed in material and methods, n≥4 for all constructs.

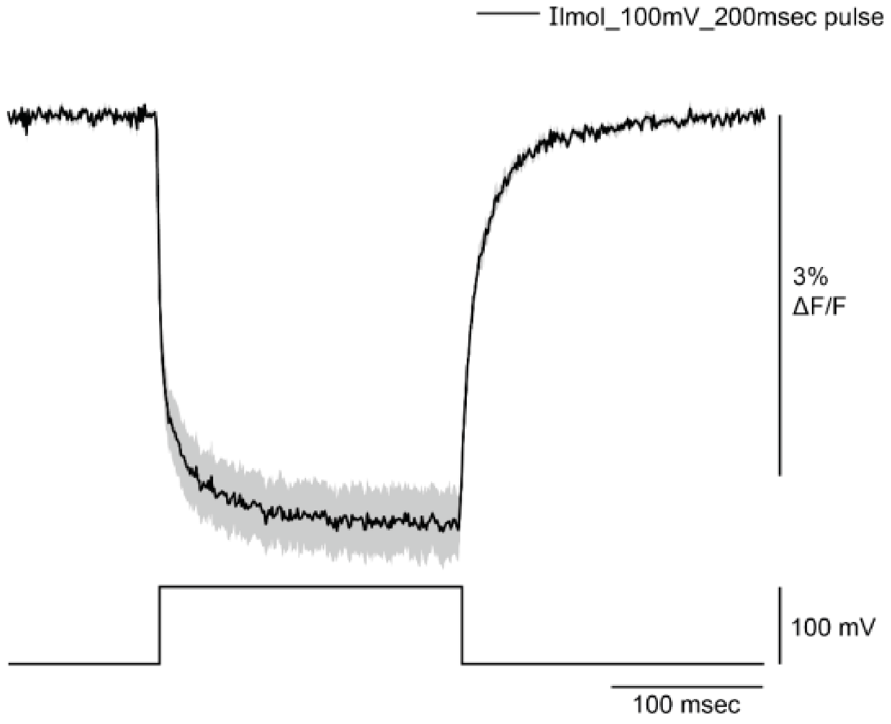

Construct	$V_{1/2}$ (mV)	τ	Fast τ (msec)	Slow τ (msec)	% fast τ
**LK11_R3_K159R (Ilmol)**	-17±2	on	2±1	28±3	73±6
		off	8±1	43±9	70±6

Supplementary Figure 2. Optical trace of Ilmol with longer time pulse did not significantly alter the kinetics or the voltage range of Ilmol.

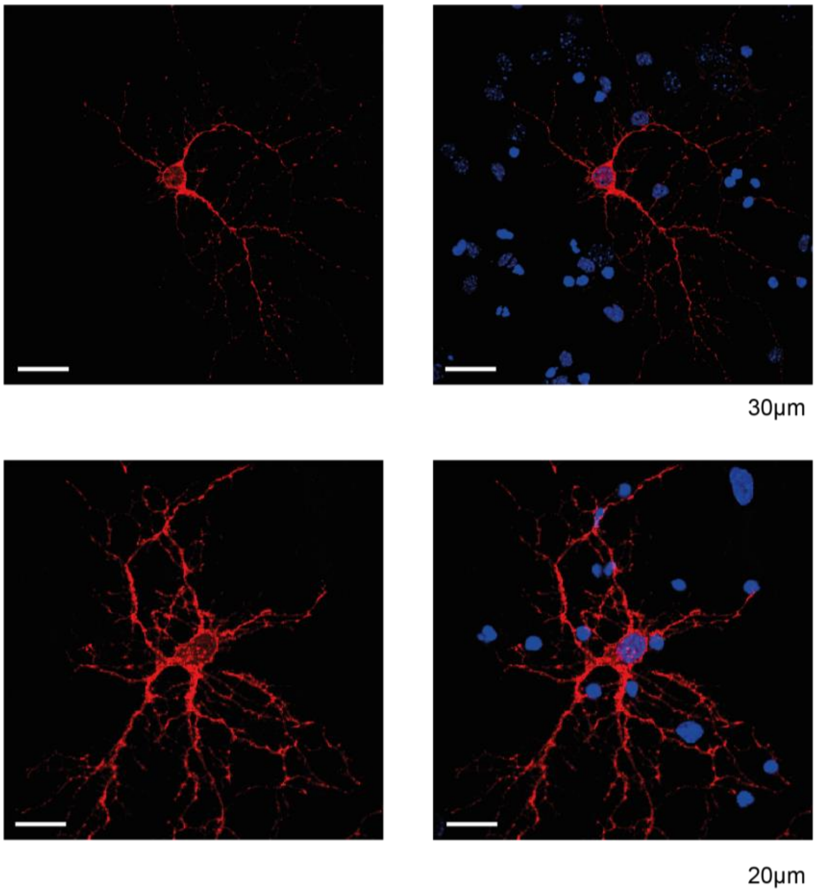

Supplementary Figure 3. Confocal image of hippocampal neurons expressing Ilmol. Blue stain in images on the right side indicates DAPI staining. The images were acquired by a confocal microscope (FV1000; Olympus, Japan). 561 nm laser was used for excitation and 595/50 nm bandpass filter was used for emission. The samples for confocal imaging were fixed with 4% paraformaldehyde solution in phosphate buffered saline adjusted at pH 7.4 and mounted with antifade mounting medium with DAPI (H-1500; Vector Laboratories, USA).

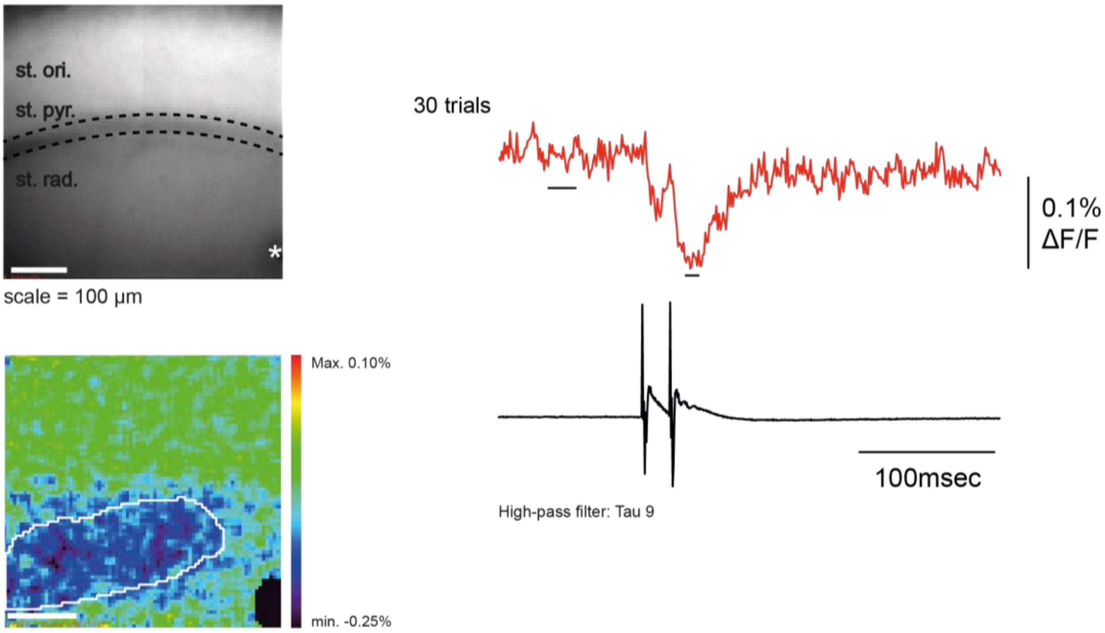

Supplementary Figure 4. Optical response of Ilmol during field stimulation in a hippocampal slice. Top image is the resting light intensity of the CA1 region of the hippocampus expressing Ilmol. The asterisk denotes the site of the stimulating electrode. Bottom image shows the heat map of neuronal activity in the slice (darkened pixels in lower right corner mask the stimulating electrode). The red trace shows the optical trace during paired pulse stimulation. The black trace is the local field potential recording. The heat map was generated by subtracting the average of 10 frames after the second stimulation from 20 frames prior to stimulation (both depicted by black dashes under the fluorescence trace). The region of interest generating the fluorescence trace on the left consists of all the pixels inside the white circle. There was no offline filtering for this trace. Sampling rate was 1000 frames per second. Light intensity was 500 mW/cm^2.
